# Pharmacodynamics, Efficacy, and Safety of Intraputaminal Eladocagene Exuparvovec Administered to Pediatric Patients With Aromatic L‐Amino Acid Decarboxylase Deficiency Using an MR‐Compatible Cannula: 48 Weeks of Follow‐Up

**DOI:** 10.1002/jimd.70151

**Published:** 2026-02-22

**Authors:** Daniel J. Curry, Phillip L. Pearl, Scellig S. D. Stone, Donald L. Gilbert, Sudhakar Vadivelu, Bruria Ben‐Zeev, Matthew Vestal, Muhammad Zafar, Chun‐Hwei Tai, Sheng‐Che Chou, Zion Zibly, Lior Ungar, Mered Parnes, Mariam Hull, Lisa Emrick, Christian Werner, Alexis Krolick, Vinay Penematsa, Antonia Wang, Rezwanur Rahman, Lee Golden, Yin‐Hsiu Chien, Paul Wuh‐Liang Hwu

**Affiliations:** ^1^ Section of Pediatric Neurosurgery, Department of Neurosurgery Baylor College of Medicine Houston Texas USA; ^2^ Boston Children's Hospital, Harvard Medical School Boston Massachusetts USA; ^3^ Division of Neurology Cincinnati Children's Hospital Medical Center Cincinnati Ohio USA; ^4^ Division of Neurosurgery Cincinnati Children's Hospital Medical Center Cincinnati Ohio USA; ^5^ Sackler School of Medicine Tel Aviv University Tel Aviv Israel; ^6^ Department of Pediatric Neurosurgery Dartmouth Geisel School of Medicine and Dartmouth Hitchcock Medical Center Lebanon New Hampshire USA; ^7^ Department of Pediatrics and Neurology Duke University Hospital Durham North Carolina USA; ^8^ Department of Neurology National Taiwan University Hospital Taipei Taiwan; ^9^ Division of Neurosurgery, Department of Surgery National Taiwan University Hospital Taipei Taiwan; ^10^ Department of Traumatology National Taiwan University Hospital Taipei Taiwan; ^11^ Department of Neurosurgery Sheba Medical Center Ramat Gan Israel; ^12^ Department of Neurosurgery Yale University Medical Center New Haven Connecticut USA; ^13^ Department of Pediatrics, Division of Neurology and Developmental Neuroscience Baylor College of Medicine Houston Texas USA; ^14^ PTC Therapeutics Germany, GmbH Frankfurt Germany; ^15^ PTC Therapeutics, Inc. Warren New Jersey USA; ^16^ Department of Medical Genetics National Taiwan University Hospital Taipei Taiwan; ^17^ Precision Medical Center, China Medical University Hospital Taichung City Taiwan

**Keywords:** AADC deficiency, eladocagene exuparvovec, gene therapy, intraputaminal administration, MR‐compatible ventricular cannula, safety

## Abstract

Aromatic ʟ‐amino acid decarboxylase (AADC) deficiency is a rare pediatric neurotransmitter disorder that typically necessitates lifelong care, and that carries a risk of childhood mortality. Eladocagene exuparvovec gene therapy is designed to restore AADC production. Study GT‐002 (NCT04903288) is a phase 2, multicenter, open‐label trial assessing the pharmacodynamics, safety, and efficacy of eladocagene exuparvovec administered to the putamen bilaterally in pediatric patients with AADC deficiency using a magnetic resonance (MR)‐compatible cannula. Patients received eladocagene exuparvovec at 1.8 × 10^11^ vector genomes via the SmartFlow MR‐compatible cannula in a single operative session. Endpoints include the change from baseline in cerebrospinal fluid homovanillic acid levels, motor milestone achievement, and safety. Here we report results from 48 weeks of follow‐up. Mean (SD) cerebrospinal fluid homovanillic acid levels increased from baseline (22.5 [32.3] nmol/L; *n* = 13) to week 48 (55.3 [45.6] nmol/L; change from baseline: 28.3 [13.7] nmol/L; *p* = 0.0003; *n* = 9), indicating *de novo* dopamine production. At baseline (*n* = 13), all patients showed severe motor developmental delay; at week 48 (*n* = 12), nine achieved full head control, four could sit unassisted, two could stand with support, and two could walk independently to a toy. Overall, 260 treatment‐emergent adverse events were reported in 13 patients; 259 were deemed unrelated and one likely unrelated to the MR‐compatible cannula. No treatment‐emergent adverse events led to study withdrawal and no deaths occurred. This study provides further evidence of the favorable pharmacodynamic, efficacy, and safety profile of eladocagene exuparvovec in children with AADC deficiency; intraputaminal administration using an MR‐compatible cannula was well tolerated. Study GT‐002 (NCT04903288) provides further evidence of the favourable pharmacodynamic, efficacy and safety profile of eladocagene exuparvovec gene therapy in children with AADC deficiency over 48 weeks and demonstrates that intraputaminal administration using an MR‐compatible cannula was well tolerated, allowing for real‐time MRI confirmation of cannula placement and infusate coverage, and for accurate dosing to the putamen.

## 1 Introduction

Aromatic ʟ‐amino acid decarboxylase (AADC) deficiency (MIM #608643) is a rare, autosomal recessive pediatric neurotransmitter disorder that presents in infancy and has a wide range of debilitating symptoms that typically necessitate lifelong care [1, 2]. The presence of pathogenic dopa decarboxylase (*DDC*; HGNC:2719) variants leads to reduced activity of the AADC enzyme (EC4.1.1.28), which is responsible for the decarboxylation of ʟ‐3,4‐dihydroxyphenylalanine and 5‐hydroxytryptophan to form the neurotransmitters dopamine and serotonin, respectively [2, 3]. The subsequent marked or complete loss of the production of dopamine and other monoamine transmitters results in arrested motor and cognitive development, movement disorders, and autonomic dysfunction [1]. Patients with severe AADC deficiency do not achieve motor milestones despite essentially normal brain structure in most cases, as determined by magnetic resonance (MR) imaging [1, 3]. Furthermore, patients are at risk of dying within the first decade of life, highlighting the need for effective disease‐modifying treatments [2].

Eladocagene exuparvovec, the first‐ever gene therapy administered directly to the brain, is approved in the European Member States, Great Britain, Iceland, Israel, Liechtenstein, Northern Ireland, Norway, Brazil, Hong Kong, and Taiwan for the treatment of patients aged ≥ 18 months with a clinical, molecular, and genetically confirmed diagnosis of AADC deficiency with a severe phenotype [4, 5, 6, 7]. In the United States (US), it is approved for adult and pediatric patients with AADC deficiency [8].

Eladocagene exuparvovec is a recombinant adeno‐associated viral vector serotype 2 (AAV2) that contains the human *DDC* gene and is designed to restore AADC production, regardless of the underlying pathogenic variant(s) in the native *DDC* gene [9]. The safety and efficacy of eladocagene exuparvovec delivered by bilateral infusion into the putamen via stereotactic surgery, using a stainless steel cannula, have been demonstrated in three clinical trials (AADC‐010, phase 1/2 [NCT01395641]; AADC‐011, phase 2b [NCT02926066]; AADC‐CU/1601, compassionate‐use program) and a long‐term follow‐up study (AADC‐1602) [4, 9]. Furthermore, significant increases in cerebrospinal fluid (CSF) homovanillic acid (HVA) levels and ^18^F‐DOPA uptake have been observed in patients following eladocagene exuparvovec gene therapy [9]. HVA is a stable end product of dopamine metabolism, and CSF HVA levels are a biomarker for *de novo* dopamine production as a result of AADC activity in the brain [9, 10]. ^18^F‐DOPA is a positron‐emitting fluorine‐labeled substrate of AADC, and its uptake into the putamen reflects AADC enzyme activity of dopaminergic neurons [8].

The present study, Study GT‐002 (NCT04903288), assessed the pharmacodynamics (including early assessment of efficacy biomarkers), safety, and efficacy of eladocagene exuparvovec over 48 weeks, administered to the putamen of pediatric patients with AADC deficiency using an MR‐compatible cannula. Use of an MR‐compatible cannula allows for real‐time MRI confirmation of cannula placement and infusate coverage, and for accurate dosing to regions of interest in the brain, bypassing the blood–brain barrier [11]. Study GT‐002 led to the approval of eladocagene exuparvovec for the treatment of AADC deficiency in the US, where the Food and Drug Administration (FDA) also granted authorization for use of the SmartFlow MR‐compatible ventricular cannula for intraputaminal administration of eladocagene exuparvovec [8, 11, 12]. The study, which is ongoing, will assess the long‐term safety and efficacy of eladocagene exuparvovec over 5 years. Here we report results up to 48 weeks.

## 2 Methods

### 2.1 Study Design

Study GT‐002 (NCT04903288) is a phase 2, multicenter, open‐label trial of eladocagene exuparvovec in pediatric patients with AADC deficiency and is being conducted in six hospitals in Israel, Taiwan, and the USA [13]. The study consists of an 8‐week trial phase followed by a 40‐week extension phase (48 weeks in total) and an ongoing long‐term extension phase in which patients will be followed up for up to 260 weeks (5 years) after eladocagene exuparvovec administration (Figure S1). Here we report pharmacodynamic and efficacy data from the extension phase (up to 48 weeks) and available safety data through the long‐term extension phase to the cut‐off date of March 1, 2024.

The study was conducted in accordance with the ethical principles of the Declaration of Helsinki and the applicable regulatory requirements. Approvals from independent ethics committees were obtained.

### 2.2 Patients

Patients were eligible for inclusion if they were ≥ 1 to < 18 years of age, with genetically confirmed AADC deficiency with typical clinical characteristics and decreased AADC enzyme activity in the plasma. It was also required that patients had a closed cranium that was sufficiently developed without defect, as determined by the neurosurgeon upon physical examination, to allow safe placement of the stereotactic head frame or bone fiducials for stereotactic surgery. Patients with an anti‐AAV2 neutralizing antibody titer > 1:1200 were not eligible to participate. Written consent to enroll in the study was provided by the patients' parents/legal guardians. Eligibility criteria are described in full in the Appendix S1.

### 2.3 Procedures

Patients received eladocagene exuparvovec at 1.8 × 10^11^ vector genomes by bilateral infusion into the putamen via the SmartFlow MR‐compatible ventricular cannula (ClearPoint Neuro Inc., Solana Beach, California, USA) in a single operative session. Patients were permitted to continue standard‐of‐care treatments for AADC deficiency as needed during the trial (dopamine agonists, monoamine oxidase inhibitors, pyridoxine, or other forms of vitamin B6).

### 2.4 Outcomes

Pharmacodynamic endpoints measured at 8 weeks and 48 weeks included change from baseline in: CSF HVA levels (primary endpoint); putaminal‐specific ʟ‐6‐[^18^F] fluoro‐3,4‐dihydroxyphenylalanine (^18^F‐DOPA) uptake, evaluated by positron emission tomography (PET) analysis; and CSF neurotransmitter metabolites 5‐hydroxyindoleacetic acid (5‐HIAA) and 3‐*O*‐methyldopa (3‐OMD). CSF neurotransmitter levels were measured using a validated high‐performance liquid chromatography with tandem mass spectrometry method [14].

Efficacy endpoints at week 48 included: attainment of key motor milestones (e.g., head control, sitting, standing, and walking); motor development as measured using the Peabody Developmental Motor Scales‐Second Edition (PDMS‐2); cognitive and language development as assessed by the Bayley Scales of Infant Development, third edition (Bayley‐III); and change from baseline in body weight and symptoms common to AADC deficiency (e.g., hypotonia). Further details on PDMS‐2 and Bayley‐III are described in the Appendix S1.

The overall safety profile of eladocagene exuparvovec was assessed by treatment‐emergent AEs (TEAEs), neurological examinations, MR imaging, and clinical laboratory tests. TEAEs were defined as AEs with a start date after receiving eladocagene exuparvovec. TEAEs were coded using MedDRA version 26.0, and the number and proportion of patients reporting TEAEs were summarized by system organ class and by preferred term. Summaries were also provided for TEAEs of special interest, for each severity grade, and for the relationship to the MR‐compatible cannula device, to gene therapy, to the neurosurgical procedure, and to ^18^F‐DOPA. Viral shedding was assessed in blood, CSF, and urine to identify any possible risk of exposure to those who come in contact with a patient. Measurements of anti‐AAV2 antibodies (both neutralizing and immunoglobulin G titers) and T‐cell response were performed to assess the immune response to eladocagene exuparvovec.

### 2.5 Statistical Analysis

No formal statistical hypothesis testing was carried out, and the sample size was not based on statistical consideration; a minimum of three participants was planned to be included in the assessment of HVA levels at week 8 and safety of the MR‐compatible cannula for administering eladocagene exuparvovec to pediatric patients.

The pharmacodynamic population comprised all patients enrolled in the study who received eladocagene exuparvovec and had both baseline and at least one post‐baseline value of at least one pharmacodynamic variable; this population was used for the analyses of pharmacodynamic endpoints. The safety population comprised all patients enrolled in the study who received eladocagene exuparvovec; this population was used for evaluation of safety endpoints. Safety data were reviewed by a data safety monitoring board. The efficacy population comprised all patients enrolled in the study who received eladocagene exuparvovec and had both baseline and at least one post‐baseline evaluation of at least one efficacy variable; this population was used for all efficacy analyses.

Observed values and change from baseline in CSF neurotransmitter metabolites, brain ^18^F‐DOPA uptake, PDMS‐2 total scores, Bayley‐III combined scores (cognitive scores and language scores, which included both expressive and receptive communication, were combined), body weight, and symptoms common to AADC deficiency were summarized using summary statistics (count, mean, SD, 95% confidence interval [CI], median, minimum and maximum, or count and percentage) for each time point. PDMS‐2 and Bayley‐III raw scores were used for the statistical summary and analyses statistics. The number and percentage of patients who achieved each motor milestone were summarized by time point. Comparisons with baseline for CSF neurotransmitter levels were assessed using the one‐sample *t*‐test. Changes in PDMS‐2 and Bayley‐III scores were assessed using least squares (LS) mean of change from baseline from a repeated measures model, with baseline score, and age at gene therapy as covariates and study and time as main effects; two‐sided *p*‐values are reported. Here we report pharmacodynamic and efficacy data up to 48 weeks and available safety data through the long‐term extension phase (cut‐off date: March 1, 2024).

## 3 Results

Between 2021 and 2023, 16 children were screened for inclusion, of whom two failed screening with an anti‐AAV2 antibody titer > 1:1200, and one had parental consent withdrawn before the baseline visit. A total of 13 children (aged 16–129 months) with AADC deficiency received eladocagene exuparvovec in accordance with the study protocol. The first patient received treatment on July 21, 2021. All 13 patients completed the 8‐week trial phase, and 12 patients completed the 40‐week extension phase (48 weeks in total). After the 8‐week assessment, one patient had withdrawal of consent to participate in the study owing to not being able to complete in‐person visits; therefore, no data were collected after the 8‐week visit for this patient. Currently, 10 patients remain enrolled in the long‐term extension phase; one patient did not enroll in the long‐term extension at the decision of their parents/legal guardians and one patient withdrew consent after 71 weeks of follow‐up (Figure S2). The mean duration of follow‐up was 72.7 weeks at data cut‐off. Individual patient characteristics are shown in Table 1. Twelve different *DDC* variants were reported in the 13 patients.

**TABLE 1:** Patient demographics and baseline characteristics.

Patient	Age at gene therapy (months); sex^a^	Weight (kg)	*DDC* genotype^b^	Pre‐treatment symptoms	History of oculogyric crisis (yes/no)	Baseline motor milestone achievement	Duration of follow‐up (weeks)
1	129; M	27.6	c.260C>T; c.286G>A	+++ hypotonia +++ limb dystonia	Yes	None	71 (discontinued)
2	33; F	13.8	c.714+4A>T; c.714+4A>T	+++ hypotonia + limb dystonia	Yes	None	101
3	31; F	13.7	c.1297dup; c.714+4A>T	+++ hypotonia ++ limb dystonia	Yes	None	84
4	27; M	10.9	c.714+4A>T; c.1297dup	+ hypotonia ++ limb dystonia	Yes	None	109
5	60; M	12.8	c.242C>T; c.242C>T	+++ hypotonia +++ limb dystonia	Yes	None	104
6	70; F	11.5	c.714+4A>T; c.714+4A>T	+++ hypotonia + limb dystonia	Yes	None	73 (discontinued)
7	46; M	17.3	c.568_569insCGATC; c.863T>C	+++ hypotonia ++ limb dystonia	Yes	None	80
8	20; F	8.7	c.714+4A>T; c304G>A	++ hypotonia ++ limb dystonia	Yes	Partial head control	48
9	16; M	7.6	c.367G>A; c.1234C>T	+ hypotonia ++ limb dystonia	Yes	None	72
10	41; F	8.8	Ex11_12del; c.557A>G	+++ hypotonia − limb dystonia	Yes	None	23 (discontinued)
11	31; F	9.6	c.714+4A>T; c.714+4A>T	+++ hypotonia − limb dystonia	Yes	None	60
12	51; M	11.7	c.304G>A; c.304G>A	++ hypotonia + limb dystonia	Yes	Sitting with assistance	48
13	33; F	9.0	c.714+4A>T; c.1297dup	+++ hypotonia + limb dystonia	Yes	None	72

*Note:* +++ = severe. ++ = moderate. + = mild. − = absent.

Abbreviations: DDC, dopa decarboxylase; F, female; M, male.

^a^ Sex was reported by the patients' parents/legal guardians.

^b^ *DDC* reference sequence: NM_001082971.1 (*DDC*).

**FIGURE 1:**
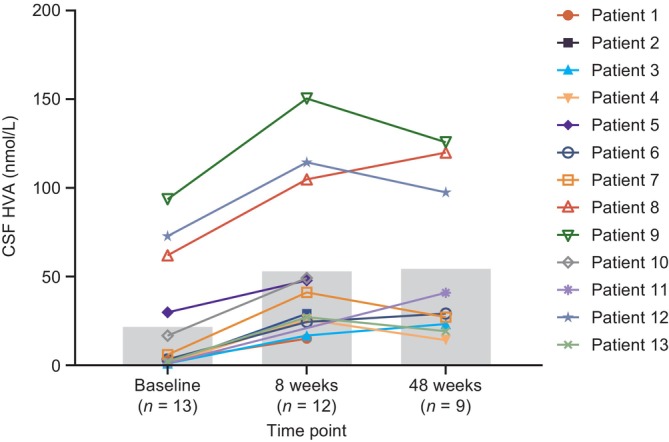
CSF HVA levels recorded for individual patients at baseline, 8 weeks, and 48 weeks following eladocagene exuparvovec gene therapy (pharmacodynamic population). Values noted as < 2.0 nmol/L are shown here as 1.0 nmol/L. The gray bars show the mean CSF HVA level at each time point. The following mean CSF HVA levels were recorded: Baseline, 22.5 (SD: 32.3) nmol/L; 8 weeks, 53.9 (SD: 44.4) nmol/L (*p* < 0.0001 vs. baseline); 48 weeks, 55.3 (SD: 45.6) nmol/L (*p* = 0.0003 vs. baseline). CSF, cerebrospinal fluid; HVA, homovanillic acid.

Low CSF HVA levels (mean: 22.5 [SD: 32.3] nmol/L; *n* = 13), consistent with a diagnosis of AADC deficiency [1], were observed at baseline. At 8 weeks after gene therapy, mean CSF HVA levels were significantly increased to 53.9 (SD: 44.4) nmol/L (mean change from baseline: 29.5 [95% CI: 21.3–37.8] nmol/L; *p* < 0.0001; *n* = 12); these increases were sustained 48 weeks after gene therapy, with mean levels of 55.3 (SD: 45.6) nmol/L (mean change from baseline: 28.3 [95% CI: 17.8–38.8] nmol/L; *p* = 0.0003; *n* = 9; Figure 1). When baseline HVA measurements were taken, 69.2% of patients (*n* = 9/13) were receiving concomitant standard‐of‐care medication for AADC deficiency, including anticholinergic or dopaminergic agents, and pyridoxine or pyridoxal phosphate. When HVA measurements were taken at 8 weeks and 48 weeks, 66.7% of patients (*n* = 8/12) and 22.2% of patients (*n* = 2/9) were receiving concomitant medication, respectively.

**FIGURE 2:**
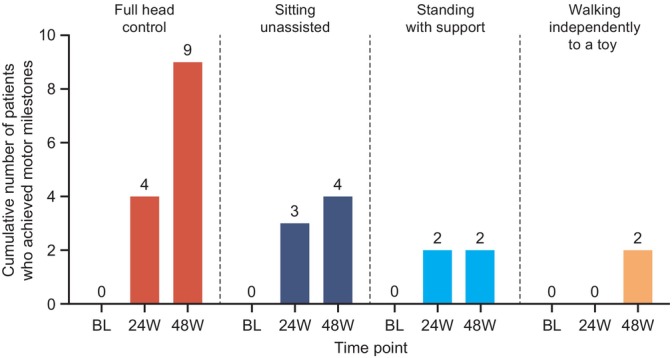
Cumulative motor milestone achievement at baseline, 24 weeks, and 48 weeks following eladocagene exuparvovec gene therapy (efficacy population). The cumulative number of patients who achieved a PDMS‐2 score of 1 (indicating emergence of the motor milestone) or 2 (indicating mastery of the motor milestone) at each time point is shown. Number of patients included at each time point: BL, *n* = 13; 24 weeks, *n* = 11; 48 weeks, *n* = 12. BL, baseline; PDMS‐2, Peabody Developmental Motor Scales‐Second Edition; W, week.

**FIGURE 3:**
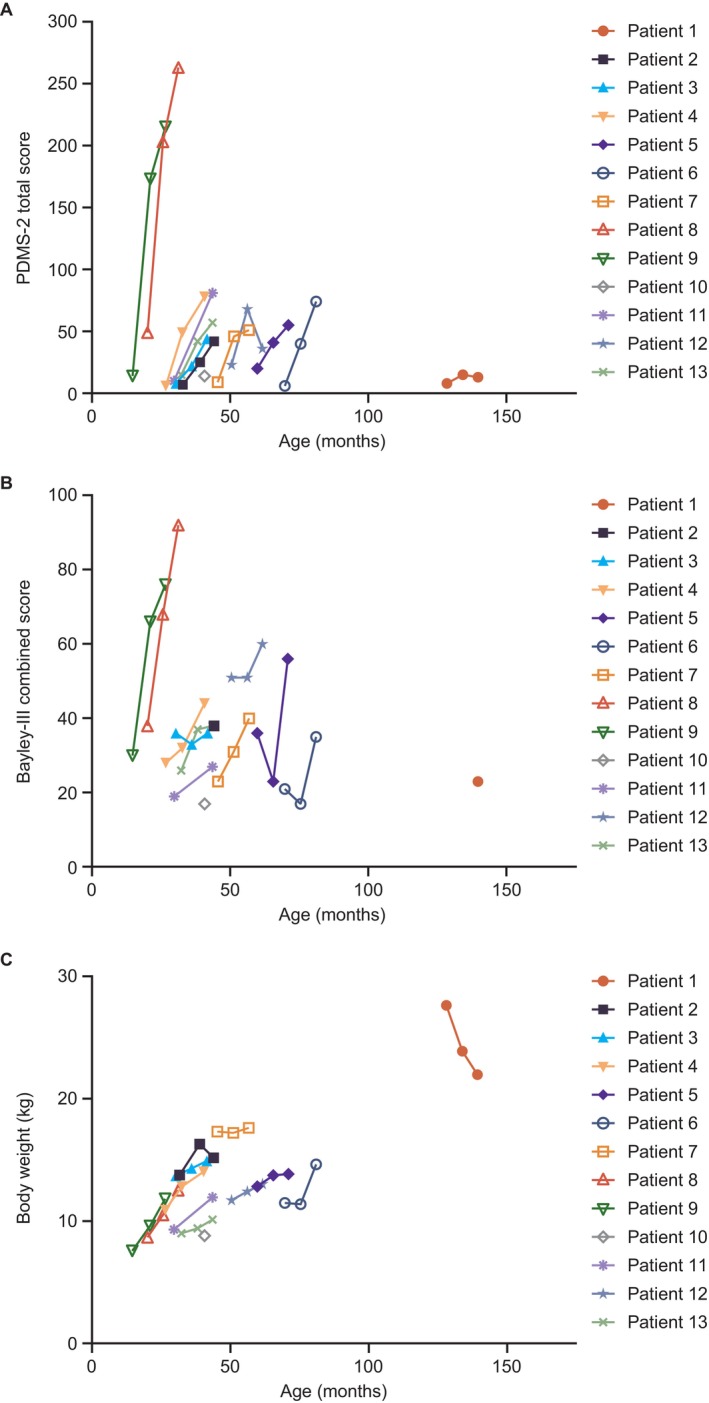
Efficacy endpoints for each patient by age following eladocagene exuparvovec gene therapy, (A) PDMS‐2 total scores, (B) Bayley‐III combined scores (combined cognitive and language scores), and (C) body weight (efficacy population). PDMS‐2, Peabody Developmental Motor Scales‐Second Edition.

At baseline, all patients showed severe motor developmental delay: as assessed by the PDMS‐2, only one patient had partial head control (patient 8), and only one patient could sit with assistance (patient 12). Among the 12 patients who reached week 48, nine achieved full head control, four could sit unassisted, two could stand with support, and two could walk independently to a toy (Figure 2). Patients 8 and 9 were able to walk backwards using a normal stride at 48 weeks; this was the highest achieved motor milestone recorded in this study at week 48. Patients 3 and 13 achieved partial head control only, after a follow‐up duration of 336 days and 335 days, respectively. Patient 2 had not achieved any motor milestones by week 48 but achieved full head control and sitting with assistance after a follow‐up duration of 626 days (data not shown). The two youngest patients (patients 8 and 9, both aged < 24 months at gene therapy) appeared to show the fastest and most pronounced improvements in motor development (Figure 3A).

The mean PDMS‐2 total score was 14.5 (SD: 11.7) at baseline (*n* = 13). At 48 weeks (*n* = 12), the mean had increased to 84.1 (SD: 75.5), corresponding to an increase of 69.5 (95% CI: 26.4–112.6) and a LS mean for change from baseline of 68.4 (95% CI: 38.6–98.1; *p* = 0.0004). At baseline (*n* = 11), the mean Bayley‐III combined score was 29.5 (SD: 10.1) and at 48 weeks (*n* = 12), the mean was 47.1 (SD: 20.4), corresponding to an increase of 19.6 (95% CI: 7.4–31.8) and an LS mean for change from baseline of 18.9 (95% CI: 8.2–29.5; *p* = 0.003). As was observed for motor development, patients who were younger at gene therapy administration appeared to show the fastest and most pronounced improvements in Bayley‐III combined score (Figure 3B). Mean body weight increased from baseline to week 48 (Figure 3C, Appendix S1).

All patients (*n* = 13/13) had hypotonia at baseline; at week 48, 16.7% of patients (*n* = 2/12) had no symptoms of hypotonia. The severity of hypotonia also improved following eladocagene exuparvovec: 69.2% of patients (*n* = 9/13) had severe hypotonia at baseline, whereas 9.1% of patients (*n* = 1/11) and 8.3% of patients (*n* = 1/12) had severe hypotonia at week 8 and week 48, respectively (Figure S3).

**FIGURE 4:**
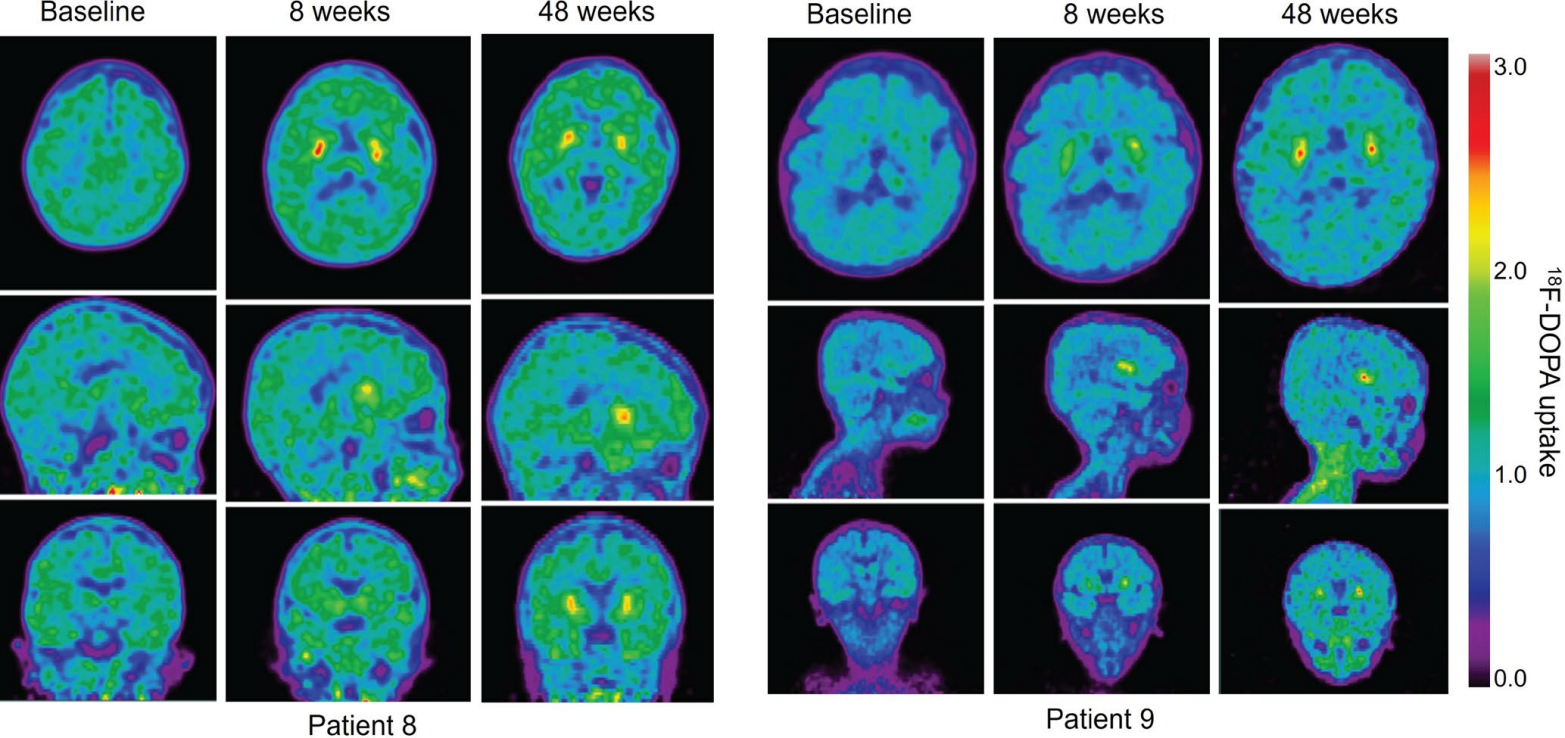
^18^F‐DOPA uptake in PET images at baseline, 8 weeks, and 48 weeks following eladocagene exuparvovec gene therapy in patient 8 and patient 9. Axial (top row), sagittal (middle row), and coronal (bottom row) PET images for patient 8 and patient 9 are representative for the study population. ^18^F‐DOPA, L‐6‐[^18^F] fluoro‐3,4‐dihydroxyphenylalanine; PET, positron emission tomography.

Minimal dopamine production was detected at baseline (mean bilateral putaminal‐specific uptake of ^18^F‐DOPA: 0.098 [SD: 0.074]; *n* = 13). At 8 weeks after gene therapy, the mean bilateral putaminal‐specific uptake of ^18^F‐DOPA was 0.343 (SD: 0.040), representing a significant increase from baseline of 271% (mean change from baseline: 0.243 [95% CI: 0.190–0.297]; *p* < 0.0001; *n* = 12; Figure S4A). At 48 weeks after gene therapy, the mean bilateral putaminal‐specific ^18^F‐DOPA uptake was 0.343 (SD: 0.113), representing a significant sustained increase from baseline of 296% (mean change from baseline: 0.253 [95% CI: 0.167–0.339]; *p <* 0.0001; *n* = 10). The significant increase in ^18^F‐DOPA uptake in the putamen was visualized in PET images taken at 8 weeks and 48 weeks after gene therapy (Figure 4). CSF 5‐HIAA and 3‐OMD levels were not significantly changed throughout the study (Appendix S1, Figure S4B,C).

At data cut‐off (mean follow‐up: 72.7 weeks), 260 TEAEs were reported in 13 patients (Table 2), of which 259 were deemed unrelated and one likely unrelated to the MR‐compatible cannula. The majority of TEAEs were determined to be unrelated to treatment and resolved at data cut‐off. Pneumonia, hypoxia, and COVID‐19 were the only severe or life‐threatening TEAEs reported in more than a single patient. No TEAEs led to withdrawal from the study. TEAEs recorded in > 50% of patients were pyrexia (*n* = 11/13; 84.6%), dyskinesia (*n* = 10/13; 76.9%), and diarrhea (*n* = 8/13; 61.5%).

**TABLE 2:** Summary of TEAEs and SAEs throughout the full follow‐up period (safety population).

Adverse event category	Patients (*N* = 13)
Number of TEAEs	260
Patients with ≥ 1 TEAE, *n* (%)	13 (100)
Most frequent TEAEs^a^, *n* (%)	
Pyrexia	11 (84.6)
Dyskinesia	10 (76.9)
Diarrhea	8 (61.5)
Cough	6 (46.2)
Dermatitis diaper	5 (38.5)
Pneumonia	5 (38.5)
Anemia	4 (30.8)
COVID‐19	4 (30.8)
Hypotension	4 (30.8)
Influenza	4 (30.8)
Nasopharyngitis	4 (30.8)
Upper respiratory tract infection	4 (30.8)
Vomiting	4 (30.8)
TEAE leading to withdrawal, *n* (%)	0 (0)
TEAE relationship to surgical device, *n* (%)	
Unrelated	13 (100)
Unlikely	1 (7.7)
Possible	0 (0)
Probable	0 (0)
TEAE relationship to neurosurgical procedure, *n* (%)	
Unrelated	13 (100)
Unlikely	3 (23.1)
Possible	6 (46.2)
Probable	5 (38.5)
TEAE relationship to gene therapy, *n* (%)	
Unrelated	13 (100)
Unlikely	7 (53.8)
Possible	6 (46.2)
Probable	9 (69.2)
Patients with TEAE related to ^18^F‐DOPA, *n* (%)	1 (7.7)
Maximum TEAE severity, *n* (%)	
Mild	13 (100)
Moderate	12 (92.3)
Severe	9 (69.2)
Life‐threatening	6 (46.2)
Fatal	0 (0)
Patients with ≥ 1 SAE, *n* (%)	9 (69.2)
Most frequent serious TEAEs^b^, *n* (%)	
Pneumonia	4 (30.8)
COVID‐19	2 (15.4)
SAEs leading to death, *n* (%)	0 (0)
Patients with SAE related to gene therapy, *n* (%)	
Unrelated	9 (69.2)
Unlikely	2 (15.4)
Possible	0 (0)
Probable	1 (7.7)

Abbreviations: ^18^F‐DOPA, L‐6‐[^18^F] fluoro‐3,4‐dihydroxyphenylalanine; SAE, serious adverse event; TEAE, treatment‐emergent adverse event.

^a^ TEAEs that occurred in ≥ 4 patients.

^b^ Serious TEAEs that occurred in ≥ 1 patient.

Forty‐eight TEAEs in 10 patients were considered possibly or probably related to eladocagene exuparvovec; dyskinesia was the most common event, and the majority were mild or moderate, with one severe event. Twenty‐one TEAEs in eight patients were considered possibly or probably related to the neurosurgical procedure, with the most common being pyrexia. One TEAE (pyrexia) was considered possibly or probably related to ^18^F‐DOPA.

In total, 21 serious TEAEs were recorded in nine patients; only two events (both dyskinesia) experienced by one patient were considered related to eladocagene exuparvovec. One patient experienced two serious TEAEs of seizures, which were severe. No deleterious immune responses were observed, and there were no deaths during the study.

Protocol‐specified TEAEs of special interest included dyskinesia, events related to the neurosurgical procedure and CSF leaks. No CSF leaks were reported, and no evidence of leaks was found on brain imaging assessments. Ten of the 13 patients experienced dyskinesia following eladocagene exuparvovec (16 events in total). The median time to onset of the first episode of dyskinesia was 27.5 days after administration and the median (minimum, maximum) duration of dyskinesia was 104.5 (11, 363) days; at data cut‐off, 12/16 events have resolved. Table 3 reports the presence and severity of dyskinesia, along with changes in CSF HVA levels and motor milestone achievement for individual patients.

**TABLE 3:** Presence and severity of dyskinesia after eladocagene exuparvovec gene therapy, CSF HVA levels before and after eladocagene exuparvovec gene therapy and achievement of motor milestones for individual patients.

Patient	Age at gene therapy (months); sex	Dyskinesia (yes/no)—severity	Duration of dyskinesia (days)	Baseline CSF HVA (nmol/L)	Week 48 CSF HVA (nmol/L)	Highest motor milestone achieved at 48 weeks^a^
1	129; M	Yes—moderate	311	2.6	NA	1—full head control
2	33; F	Yes—moderate	168	< 2.0	NA	NA^b^
3	31; F	Yes—mild	309	< 2.0	23.3	1—partial head control
4	27; M	Yes—mild	363	< 2.0	14.2	2—sitting unassisted
5	60; M	Yes—mild	311	29.9	NA	2—full head control
6	70; F	Yes—mild	102	3.3	29.2	1—sitting with assistance
7	46; M	Yes—moderate/mild	55/169	6.0	27.2	2—sitting with assistance
8	20; F	Yes—moderate/mild	55/107	62.0	120.0	2—walking backwards using normal stride
9	16; M	Yes—mild	23	93.7	125.8	1—walking backwards using normal stride
10	41; F	No	NA	16.7	NA	NA^c^
11	31; F	No	NA	< 2.0	40.9	1—sitting with assistance
12	51; M	No	NA	72.8	97.5	2—sitting unassisted
13	33; F	Yes—mild/severe/moderate/mild	13/25/14/11	2.1	19.2	1—partial head control

Abbreviations: CSF, cerebrospinal fluid; F, female; HVA, homovanillic acid; M, male; NA; not available.

^a^ A PDMS‐2 score of 1 indicates emergence of the motor milestone, and a score of 2 indicates mastery of the motor milestone.

^b^ Patient 2 had not achieved any motor milestones at week 48.

^c^ Patient 10 withdrew from the extension phase after 23 weeks of follow‐up owing to the family being unable to do in‐person visits; therefore, HVA levels and motor milestone data were not collected at week 48.

At screening, 92.3% of patients (*n* = 12/13) had negative serum immunoglobulin G (IgG). Patient 11, who had positive anti‐AAV2 IgG antibody titers at screening and at baseline, was negative for the presence of neutralizing antibodies at baseline. By 8 weeks after administration of eladocagene exuparvovec, *n* = 12/12 patients tested positive for anti‐AAV2 IgG antibodies (one patient had missing value at week 8). The mean anti‐AAV2 antibody IgG titer at week 8 was 62200.0 (SD: 69757.5). No viral shedding was detected in post‐surgery CSF or urine samples. Quantifiable levels of virus were measured in the blood of four patients at day 3 but were below the limit of detection (3.1 copies/mL blood) by week 3.

## 4 Discussion

This ongoing study evaluates the pharmacodynamics, efficacy, and safety of eladocagene exuparvovec gene therapy administered by bilateral infusion into the putamen of pediatric patients with AADC deficiency using an MR‐compatible cannula. Results from the completed extension phase of the trial (48 weeks in total) demonstrate that in patients aged 16–129 months at administration, eladocagene exuparvovec led to a marked increase in CSF HVA levels and ^18^F‐DOPA uptake from baseline to 48 weeks after treatment. Furthermore, the acquisition of key motor milestones was observed after eladocagene exuparvovec gene therapy and patients showed cognitive and language development despite all having severe developmental delay at baseline. TEAEs were consistent with the known safety profile of eladocagene exuparvovec [4, 5, 8, 9], and administration with the MR‐compatible cannula was well tolerated.

HVA is a stable end product of dopamine metabolism, and CSF HVA levels are a biomarker for dopamine production as a result of AADC activity in the brain [9, 10]. In this study, the primary pharmacodynamic endpoint of change in CSF HVA levels from baseline was met; following eladocagene exuparvovec gene therapy, significant increases in CSF HVA levels were observed as early as 8 weeks and were sustained up to 48 weeks, indicating *de novo* and continued dopamine production. The number of patients receiving concomitant standard‐of‐care therapies decreased from baseline to week 48, suggesting that the increases in HVA at all time points were independent of concomitant use of standard‐of‐care therapies.

The assessment of pharmacodynamic endpoints 8 weeks after gene therapy administration is the earliest assessment to date, and these results suggest that the effects of eladocagene exuparvovec on dopamine production occur rapidly. The level of HVA change in this study was consistent with the magnitude of change observed in previous studies and denotes a functioning *DDC* gene and restoration of AADC enzyme activity [9].

Further evidence of increased AADC expression was indicated by the significant increases in ^18^F‐DOPA uptake observed at 8 weeks and 48 weeks after the administration of eladocagene exuparvovec. ^18^F‐DOPA crosses the blood–brain barrier, is taken up by presynaptic nigrostriatal dopaminergic neurons in the putamen and is converted by AADC to dopamine [9, 15, 16]; therefore, increased ^18^F‐DOPA putaminal uptake demonstrates *de novo* dopamine production indicative of restored AADC enzyme activity. These results are consistent with previously published results: in clinical trials, there was a statistically significant increased uptake of ^18^F‐DOPA that was sustained up to 5 years post gene therapy [9]; and in two patients who received eladocagene exuparvovec through compassionate use in Europe, increased uptake of ^18^F‐DOPA was reported at 1 month and at 1 year versus baseline [17]. Interestingly, significant and meaningful increases in CSF HVA levels and ^18^F‐DOPA uptake were observed at week 8. These increases were sustained to week 48, suggesting that it is important to monitor changes in CSF metabolites at early time points following treatment, because early increases in dopamine production following eladocagene exuparvovec administration may be indicative of the levels that will be achieved over the longer term. Similarly, it has been shown in previous studies that eladocagene exuparvovec led to increased CSF HVA levels and 18F‐DOPA uptake in the putamen 12 months after gene therapy, with levels sustained up to 5 years, consistent with the durability of motor milestone development [9].

Following treatment with eladocagene exuparvovec, no significant increases in CSF 5‐HIAA levels were observed, which is consistent with previous studies [9]. An overall numerical decrease in mean 3‐OMD levels from baseline was observed, which would be consistent with restored AADC enzyme activity; however, results were not significant and varied between patients.

Both the increased CSF HVA levels and ^18^F‐DOPA uptake, indicative of improved AADC activity, coincided with the achievement of clinically meaningful key motor milestones and cognitive and language development. In addition, PDMS‐2 total scores increased in the 48 weeks post gene therapy, corroborating the clinical meaningfulness of assessing PDMS‐2 total scores in relation to the achievement of motor milestones. Nearly all patients demonstrated improvements in motor development by 48 weeks, although the rate of achievement of milestones varied. In comparison, in a natural history cohort of 43 untreated patients with AADC deficiency with a severe phenotype, no patients had documented motor milestone achievement at a median (min, max) age of 7.2 (2, 19) years [8]. The two youngest patients in this study (patients 8 and 9; both aged < 24 months at eladocagene exuparvovec administration) had the fastest and greatest improvements in motor development. Both showed rapid increases in PDMS‐2 total scores (to > 150) within 24 weeks and could walk backwards using a normal stride by week 48. These results are consistent with findings from previous clinical trials of eladocagene exuparvovec in patients with AADC deficiency [9]; all patients showed increases in PDMS‐2 total scores from baseline, and patients who received eladocagene exuparvovec at a younger age demonstrated a faster treatment response and appeared to reach a higher level of motor development by 8 years post gene therapy [4, 9]. The younger patients in this study who responded well to eladocagene exuparvovec in terms of motor development also showed the greatest improvements in Bayley‐III combined score, indicative of improved cognitive and language development. Overall, these data suggest that the response to eladocagene exuparvovec may be greater when treatment is received at a younger age, perhaps owing to the worsening of complications associated with this disease over time [9, 18]. This highlights the importance of increasing disease awareness among general pediatricians and the need for early and prompt diagnoses. However, the oldest patient in the study (aged 129 months at administration) still showed improvements in hypotonia and motor development and had achieved full head control at 48 weeks, further demonstrating the benefits of eladocagene exuparvovec in older patients. This finding is also confirmed by results in two children who received eladocagene exuparvovec at > 10 years old through a compassionate use program in Europe and showed improvements in motor, cognitive, and behavioral function 18 months after gene therapy [17]. It is important to note that motor milestone acquisition in patients with AADC deficiency may occur gradually over an extended period [19], and long‐term follow‐up from three previous eladocagene exuparvovec gene therapy trials showed continued improvements over time [9]. Therefore, a follow‐up of longer than 48 weeks in the current study would likely capture further acquisition of motor milestones in patients of all ages.

The most common initial symptoms of AADC deficiency are often nonspecific, such as hypotonia and developmental delay [1, 20, 21]. Neurological symptoms also include oculogyric crises episodes, dystonia, and loss of muscle function [1, 2]. All patients included in this study had hypotonia at baseline. The prevalence and severity of hypotonia improved as early as 8 weeks, and improvements continued throughout the 48 weeks of follow‐up.

The most commonly reported TEAEs in this study were pyrexia and dyskinesia, consistent with the known safety profile of eladocagene exuparvovec [4, 5, 8, 9]. Dyskinesia has been attributed to dopamine receptor hypersensitivity following long‐term dopamine deficiency [3, 9], which suggests that post‐gene therapy dyskinesia could be considered an indicator of *de novo* dopamine production. However, it should be noted that there was no indication that the severity or length of dyskinesia following gene therapy was associated with the level of improvement in motor development outcomes in this study. A position statement on the rehabilitation of patients with AADC deficiency following gene therapy published in 2024 recommended that the presence of dyskinesia should not delay the start of rehabilitation [22]; similar to spontaneous movements during early infancy, the involuntary movements of body segments experienced during episodes of dyskinesia can provide an important foundation for the future development of motor function [22]. Structured outpatient follow‐up is a critical step in ensuring patient safety because transient side effects may require hospitalization and intensive care [23].

None of the TEAEs from the trial were considered related to the MR‐compatible cannula, suggesting that this has a favorable safety profile and is well tolerated (primary safety endpoint). These results support the use of the MR‐compatible cannula for the intraputaminal administration of eladocagene exuparvovec in pediatric patients.

Measurement of anti‐AAV2 antibodies was performed to assess the immune response to eladocagene exuparvovec. All patients were positive for anti‐AAV2 antibodies by week 8; however, these did not cause any safety concerns, and eladocagene exuparvovec was still efficacious. No viral shedding was detected in post‐surgery CSF or urine samples, and by week 3, levels of virus in the blood were below the limit of detection in all patients, suggesting that the risk of third‐party transmission of the virus is very low. The exclusion criteria antibody titer cut‐off of > 1:1200 in this trial was based on previous preclinical data [24]; however, more recent preclinical and clinical trial data have demonstrated that administering gene therapies into solid tissue, such as the brain, significantly reduces the chance of pre‐existing anti‐AAV antibodies negatively impacting transduction of the gene therapy, compared with systemic delivery [25, 26, 27, 28]. It is thought that delivery directly to the brain may protect the gene therapy from the impact of pre‐existing neutralizing antibodies due to the blood–brain barrier [28]. Furthermore, evidence from clinical trials of intraputaminal AAV2‐mediated gene therapy in adults with Parkinson's disease has shown that even in patients with high neutralizing antibody titers before gene therapy, therapeutic efficacy was preserved and no safety issues were reported [26, 29, 30]. Therefore, based on the substantial treatment benefit observed with eladocagene exuparvovec and the low risk of pre‐existing neutralizing antibodies, patients with pre‐existing immunity in real‐world clinical practice should be considered for eladocagene exuparvovec gene therapy.

In the 13 patients enrolled into this study, 12 different *DDC* variants were reported. Of these, 11 had previously been classified as pathogenic or likely pathogenic [31, 32]. Five patients had a homozygous variant within the *DDC* gene. Three patients were homozygous for the *DDC* founder variant c.714+4A>T, and four patients were compound heterozygous with one allele c.714+4A>T. This variant is prevalent in the Chinese population and is associated with a severe phenotype [9, 33, 34]. One patient had a novel *DDC* variant that had not been previously reported (c.568_569insCGATC). Results from this study in patients with a range of different *DDC* variants suggest that eladocagene exuparvovec may be effective regardless of the pathogenic variant, with a tolerable safety profile across variants. This is likely because the eladocagene exuparvovec vector contains cDNA for the entire *DDC* gene.

The small patient population enrolled in this trial is an inherent limitation owing to the rarity of AADC deficiency. We also acknowledge that patients in this study had severe disease at baseline; therefore, the treatment effects in patients with mild to moderate disease could not be evaluated. However, since AADC deficiency is a monogenic disease with a clearly established single molecular pathway presenting with a wide spectrum of severity [35], eladocagene exuparvovec could also be considered a potentially beneficial therapeutic option for patients with milder disease phenotypes. Another limitation was that details of post‐gene therapy rehabilitation were not collected; the importance of rehabilitation (including physical therapy and cognitive and communication training) in AADC deficiency has been highlighted previously, to maximize improvements in motor and cognitive function [22]. Variability in the level of rehabilitation received may contribute to the differences in individual outcomes that were observed. In future studies, it would be useful to collect this information to understand the impact that post‐gene therapy rehabilitation has on the efficacy of eladocagene exuparvovec in pediatric patients with AADC deficiency.

In summary, AADC deficiency is a debilitating disease, and without disease‐modifying treatment, patients experience severe developmental delays and are unlikely to gain any motor or language skills [2, 8, 20, 21]. Furthermore, patients are at risk of dying within the first decade of life, highlighting the need for effective disease‐modifying treatments [2, 20]. Eladocagene exuparvovec treats the underlying cause of AADC deficiency, and previous studies have shown that it can lead to increases in motor and cognitive function, as well as alleviating symptoms common to AADC deficiency and improving carers' quality of life [9]. This study provides further evidence of the clinically meaningful benefits of eladocagene exuparvovec in children with AADC deficiency and its potential to significantly improve patient outcomes. These benefits include pharmacodynamic evidence of *de novo* dopamine production as early as 8 weeks after administration, which was associated with improvements in motor and cognitive function, body weight, and symptoms common to AADC deficiency. These improvements were observed even though all patients had severe motor developmental delay before treatment. This study also adds to the body of evidence for the favorable long‐term safety profile of eladocagene exuparvovec in pediatric patients with AADC deficiency and demonstrates that administration of eladocagene exuparvovec using the ClearPoint SmartFlow ventricular MR‐compatible cannula was well tolerated. Based on previous studies, the full clinical benefit of eladocagene exuparvovec is not observed until beyond 48 weeks [9]; longer‐term efficacy and safety data continue to be collected as part of the long‐term extension phase.

## Author Contributions

D.J.C. and P.L.P. were study investigators and contributed to the development of the trial design, data analysis, and the conception of the manuscript. S.S.D.S., D.L.G., S.V., B.B.‐Z., M.V., M.Z., C.‐H.T., S.‐C.C., Z.Z., L.U., M.P., M.H., L.E., Y.‐H.C., and P.W.‐L.H. were study investigators and contributed to the conception of the manuscript. C.W. and A.K. contributed to the conception of the manuscript. V.P. and R.R. contributed to the development of the trial design, conduct of the study, and reviewed and verified all data. A.W. carried out the formal statistical analysis and reviewed and verified all data. L.G. contributed to the development of the study. All authors reviewed and edited the manuscript draft and approved the final version that was submitted.

## Funding

The study was funded by PTC Therapeutics Inc. The funder was involved in the design of the study, data collection, analysis, interpretation, and the decision to submit the manuscript. The authors wrote the manuscript with medical writer assistance, which was funded by PTC Therapeutics Inc. The authors confirm independence from the sponsors; the content of the article has not been influenced by the sponsors.

## Ethics Statement

The protocol, amendments, and patient informed consent forms were approved by the Institutional Review Board or Independent Ethics Committee and competent authority according to national and international regulations. Each study site had an independent, local ethics committee that granted ethical approval for the study, details of which are outlined as follows: (i) Advarra IRB, Columbia, Maryland, USA, (ii) Boston Children's Hospital IRB, Boston, Massachusetts, USA, (iii) Cincinnati Children's Hospital IRB, Cincinnati, Ohio, USA, (iv) Duke University Health System Institutional Review Board, Durham, North Carolina, USA, (v) Helsinki Committee, Sheba Medical Center, Ramat Gan, Israel, and (vi) Research Ethics Committee of National Taiwan University Hospital, Taipei, Taiwan.

## Consent

Written consent to enroll in the study was provided by the patients' parents/legal guardians.

## Conflicts of Interest

Study GT‐002 (NCT04903288) was funded by PTC Therapeutics Inc. This study utilizes AADC gene therapy, which is the subject of a licensing arrangement in place between the sub‐investigator, P.W.‐L.H., and the Sponsor, and includes the potential for royalty payments. D.J.C. is a consultant for Medtronic; has received research support from Grace Science, Neurogene, and PTC Therapeutics; and has received travel support from Aesculap and ClearPoint Neuro. P.L.P. has received research support paid to his department from Medicure (pyridoxamine‐5′‐phosphate oxidase deficiency) and PTC Therapeutics (AADC deficiency); has received speaker fees from PTC Therapeutics; and has received publication royalties from Cambridge University Press, Demos Medical Publishing/Springer, Elsevier, and UpToDate. S.S.D.S. has received consulting fees from Alcyone Therapeutics (intrathecal drug delivery), Microbot Medical (hydrocephalus), and PTC Therapeutics (Angelman syndrome gene therapy development); and has received travel support from Bionaut and ClearPoint Neuro. D.L.G. has received research support from the US Department of Defense (neurofibromatosis type 1) and the US National Institute of Mental Health (Tourette syndrome and attention‐deficit/hyperactivity disorder); has received compensation for expert testimony for the US National Vaccine Injury Compensation Program and medical opinions through Advanced Medical/Teladoc; has provided consultation services for Emalex Biosciences and PTC Therapeutics; has provided educational talks for Illumina and through his institution as a site investigator for clinical trials for Emalex Biosciences (Tourette syndrome) and Quince Therapeutics (ataxia–telangiectasia); and has received travel support from PTC Therapeutics. S.V. serves on the data safety monitoring board for Alcyone Therapeutics and on scientific advisory boards with Anuncia Medical and Longeviti. He holds a US medical device patent on intellectual property unrelated to the research presented here. B.B.‐Z. is a consultant for PTC Therapeutics. M.Z. has been an advisory board member for Acadia Pharmaceuticals, Catalyst Pharmaceuticals, Eisai, and UCB; and has served as a research investigator for Eisai, Jazz Pharmaceuticals, LivaNova, Marinus Pharmaceuticals, Medicure, Neurelis, Neurocrine Biosciences, SK Life Science, and UCB Biopharma. C.W. and A.K. were employees of PTC Therapeutics at the time of the study. V.P., A.W., R.R., and L.G. are employees of, and own stocks and shares in, PTC Therapeutics. Y.‐H.C. has served as an advisory board member for Amicus Therapeutics, Biogen, Novartis, Sanofi, and Takeda; is or was a research investigator for Biogen and Sanofi; is or was a consultant for Biogen, Novartis, and PTC Therapeutics; and has served as a speaker for Biogen, Novartis, PTC Therapeutics, and Sanofi. P.W.‐L.H. has received research grants and royalties, served as an advisory board member and consulted for PTC Therapeutics. M.V., C.‐H.T., S.‐C.C., Z.Z., L.U., M.P., M.H., and L.E. have nothing to disclose.

## Supporting information


**Appendix S1:** Supporting information.
**Supplementary Methods.** Inclusion and exclusion criteria; Efficacy endpoints.
**Supplementary Results.** 5‐HIAA levels; 3‐OMD levels; Body weight
**Figure S1:** Study design.
**Figure S2:** Trial profile.
**Figure S3:** Proportion of patients with recorded severity of hypotonia at baseline, 8 weeks, and 48 weeks following eladocagene exuparvovec gene therapy (efficacy population).
**Figure S4:** (A) Mean bilateral putaminal‐specific uptake of ^18^F‐DOPA, (B) CSF 5‐HIAA levels, and (C) CSF 3‐OMD levels recorded for each patient at baseline, 8 weeks, and 48 weeks following eladocagene exuparvovec gene therapy.

## Data Availability

Individual patient data from this trial will not be published in the public domain. Trial data will be published on ClinicalTrials.gov following conclusion of the trial.
