# Supplementary material for: Pharmacodynamics, Efficacy, and Safety of Intraputaminal Eladocagene Exuparvovec Administered to Pediatric Patients With Aromatic L‐Amino Acid Decarboxylase Deficiency Using an MR‐Compatible Cannula: 48 Weeks of Follow‐Up
Source: J Inherit Metab Dis. 2026 Feb 22;49(2):e70151. doi: 10.1002/jimd.70151 (PMC12925825; doi:10.1002/jimd.70151)
Supplement: Supplementary file 1 — Appendix S1: Supporting information. Supplementary Methods. Inclusion and exclusion criteria; Efficacy endpoints. Supplementary Results. 5‐HIAA levels; 3‐OMD levels; Body weight Figure S1: Study design. Figure S2: Trial profile. Figure S3: Proportion of patients with recorded severity of hypotonia at baseline, 8 weeks, and 48 weeks following eladocagene exuparvovec gene therapy (efficacy population). Figure S4: (A) Mean bilateral putaminal‐specific uptake of 18F‐DOPA, (B) CSF 5‐HIAA levels, and (C) CSF 3‐OMD levels recorded for each patient at baseline, 8 weeks, and 48 weeks following eladocagene exuparvovec gene therapy. [file JIMD-49-0-s001.pdf]

Supplementary Materials

Supplementary appendix

Methods....2

Inclusion criteria....2
Exclusion criteria....3
Efficacy endpoints....4

Results....5

5-HIAA levels....5
3-OMD levels....5
Body weight....6

References....6

Supplementary figures....7

Methods

Inclusion criteria

1. Paediatric patients must have genetically confirmed aromatic ʟ-amino acid decarboxylase (AADC) deficiency with typical clinical characteristics and decreased AADC enzyme activity in plasma.
2. Aged ≥1 to <18 years.
3. A cranium sufficiently developed to allow placement of the ClearPoint system for stereotactic surgery, as determined by the neurosurgeon upon examination.
4. Persistent neurological defects secondary to AADC deficiency despite standard medical therapy (dopamine agonists, monoamine oxidase inhibitors, pyridoxine, or other forms of vitamin B6) in the opinion of the investigator.
5. Unable to ambulate independently (with or without assistive device).
6. Baseline haematology, chemistry and coagulation values within the normal paediatric laboratory value ranges, unless in the investigator's opinion the out-of-range values are not clinically significant with respect to the patient's suitability for surgery.
7. The patient must test negative for COVID-19 a maximum of 72 hours before receiving gene therapy.
8. The patient must be on stable dosage for 3 months before baseline for all medications related to treatment of AADC deficiency, including dopamine agonists, monoamine oxidase inhibitors, anticholinergic drugs and vitamin B6.
9. Female patients of childbearing potential must have a negative pregnancy test result at screening and baseline and agree to abstinence or use of a double-

barrier form of contraception for the duration of the study following discharge from the hospital (acceptable methods will be determined by the site).

10. Male patients sexually active with females of childbearing potential must agree to use a barrier method of birth control for the duration of the study following discharge from the hospital.
11. Parent(s)/legal guardian(s) with custody of the patient must agree to comply with the requirements of the study, including the need for frequent and prolonged follow-up.
12. Parent(s)/legal guardian(s) with custody of the patient must give their consent for the patient to enrol in the study.

Exclusion criteria

1. The patient has other significant medical or neurological conditions that would create an unacceptable operative or aesthetic risk.
2. Patients with pyridoxine 5'-phosphate oxidase or tetrahydrobiopterin deficiency.
3. Contraindication for imaging studies (computed tomography scan, positron emission tomography, or magnetic resonance imaging), including sedation limitations or metal that would interfere with brain magnetic resonance imaging.
4. Anti-adeno-associated viral vector serotype 2 (AAV2) antibody titre >1:1200 or >1 optical density value by enzyme-linked immunosorbent assay.
5. Patients who have received treatment with other experimental therapies within the last 24 weeks before planned gene therapy administration, or any treatment ever with a gene therapy.

6. Evidence of a clinically active infection.
7. Female patients who are pregnant or breastfeeding.

Efficacy endpoints

PDMS-2

The Peabody Developmental Motor Scales-Second Edition (PDMS-2) assesses the gross and fine motor skills of children between 0 and 71 months of age. It is scored on a scale of 0–2, with 0 being equivalent to the motor milestone not being met, 1 being an emerging skill and 2 being mastery of the motor milestone.[1] A score of 1 or 2 is classed as achieving the milestone.

Bayley-III

Bayley-III is a standardized assessment of developmental functioning for children between 1 and 42 months of age. It enables the assessment of five behaviour domains: cognitive, language, motor, social-emotional and adaptive functioning. In addition to scoring, assessors had the opportunity to include qualitative comments during the Bayley-III assessment.[2] Since the Bayley-III motor scale has a lot of overlap with PDMS-2, only the cognitive and language scales from the Bayley-III were used in this study; therefore, the total of the cognitive and language scores are referred to as the combined Bayley-III score. A higher score indicates better development.

Results

5-HIAA levels

At baseline, low cerebrospinal fluid (CSF) levels of 5-hydroxyindoleacetic acid (5-HIAA) were recorded (mean: 5.2 [SD: 12.1] nmol/L; n=13); one patient had a CSF 5-HIAA level of 45.0 nmol/L at baseline, which was substantially higher than the other patients. As expected, CSF 5-HIAA levels were not significantly changed after gene therapy, when measured at 8 weeks (mean: 6.0 [SD: 13.4] nmol/L; mean change from baseline: 0.5 [95% CI: −0.1, 1.0] nmol/L; p=0.1103; n=12) and at 48 weeks (mean: 5.3 [SD: 10.3] nmol/L; mean change from baseline: −1.3 [95% CI: −4.5, 1.9] nmol/L; p=0.3858; n=9; figure S4B).

3-OMD levels

High CSF levels of 3-*O*-methyldopa (3-OMD) were recorded at baseline (mean: 693.1 [SD: 272.5] nmol/L; n=13). CSF 3-OMD levels were not significantly reduced at 8 weeks after gene therapy (mean: 698.3 [SD: 216.6] nmol/L; mean change from baseline: −36.9 [95% CI: −135.8, 62.1] nmol/L; p=0.4260; n=11). At 48 weeks, CSF 3-OMD levels were numerically but not significantly reduced (mean: 573.9 [SD: 170.2] nmol/L; mean change from baseline: −54.8 [95% CI: −282.5, 172.9] nmol/L; p=0.5942; n=9; figure S4C).

Body weight

At baseline (n=13), mean body weight was 12.5 (SD: 5.3) kg and at week 48 it was 14.3 (SD: 3.1) kg (n=12), representing a mean increase of 1.5 (95% CI: −0.2, 3.1) kg. In total, 91.7% of patients (n=11/12) had a gradual increase in body weight over 48 weeks; only one patient (patient 1 – the oldest patient included in the study) experienced a decrease in body weight over the 48 weeks following gene therapy (figure 3C).

References

1. Folio MR, Fewell RR. *Peabody Developmental Motor Scales: Examiner's Manual*. 2nd ed. Austin, TX: Pro-Ed; 2000.
2. Weiss LG, Oakland T, Aylward GP. *Bayley-III Clinical Use and Interpretation.* 1st ed. Amsterdam: Academic Press; 2010.

Supplementary figures

Figure S1. Study design.

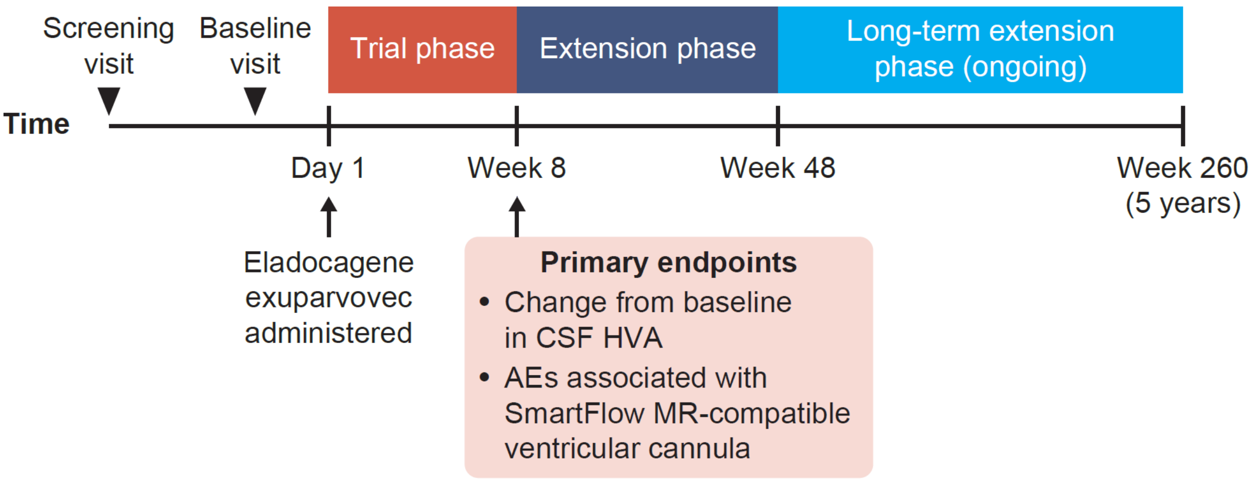

The trial phase, which assessed the pharmacodynamics of eladocagene exuparvovec and the safety of the SmartFlow MR-compatible ventricular cannula, was completed 8 weeks after administration of eladocagene exuparvovec. The extension phase, designed to capture additional clinical information (including changes in motor development), was completed 48 weeks after eladocagene exuparvovec administration. The long-term extension phase is ongoing and will follow patients for up to 260 weeks (5 years) after administration to capture long-term safety and efficacy data.

AE, adverse event; CSF, cerebrospinal fluid; HVA, homovanillic acid; MR, magnetic resonance.

Figure S2. Trial profile.

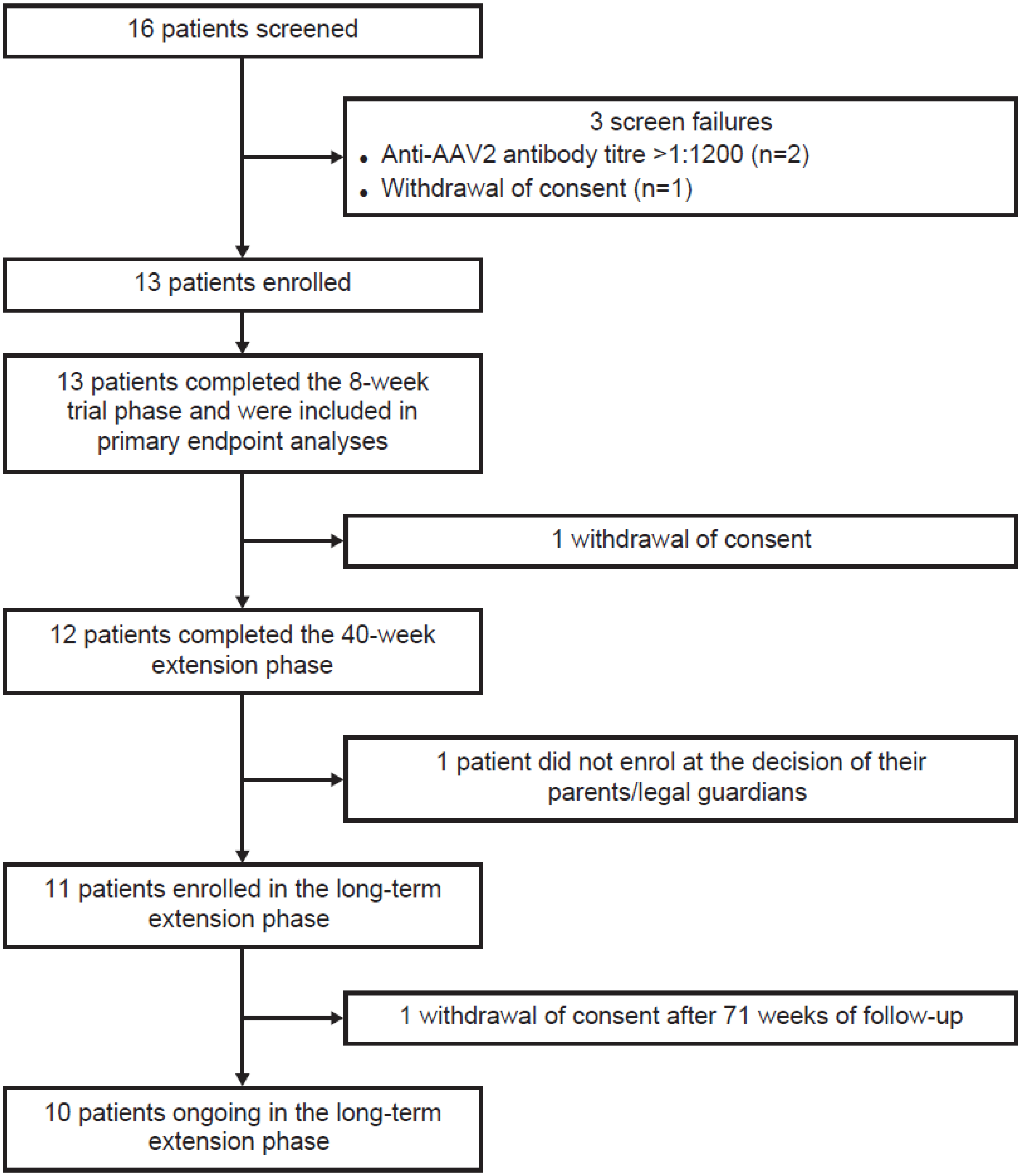

AAV2, adeno-associated viral vector serotype 2.

Figure S3. Proportion of patients with recorded severity of hypotonia at baseline, 8 weeks and 48 weeks following eladocagene exuparvovec gene therapy (efficacy population).

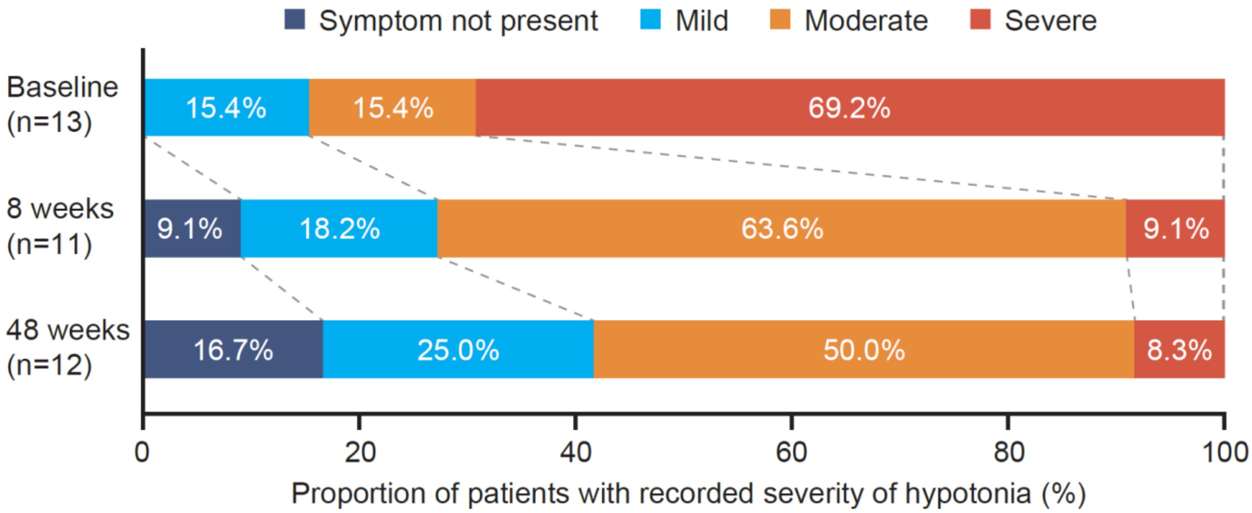

Figure S4. (A) Mean bilateral putaminal-specific uptake of ^{18}F-DOPA, (B) CSF 5-HIAA levels and (C) CSF 3-OMD levels recorded for each patient at baseline, 8 weeks and 48 weeks following eladocagene exuparvovec gene therapy (pharmacodynamic population).

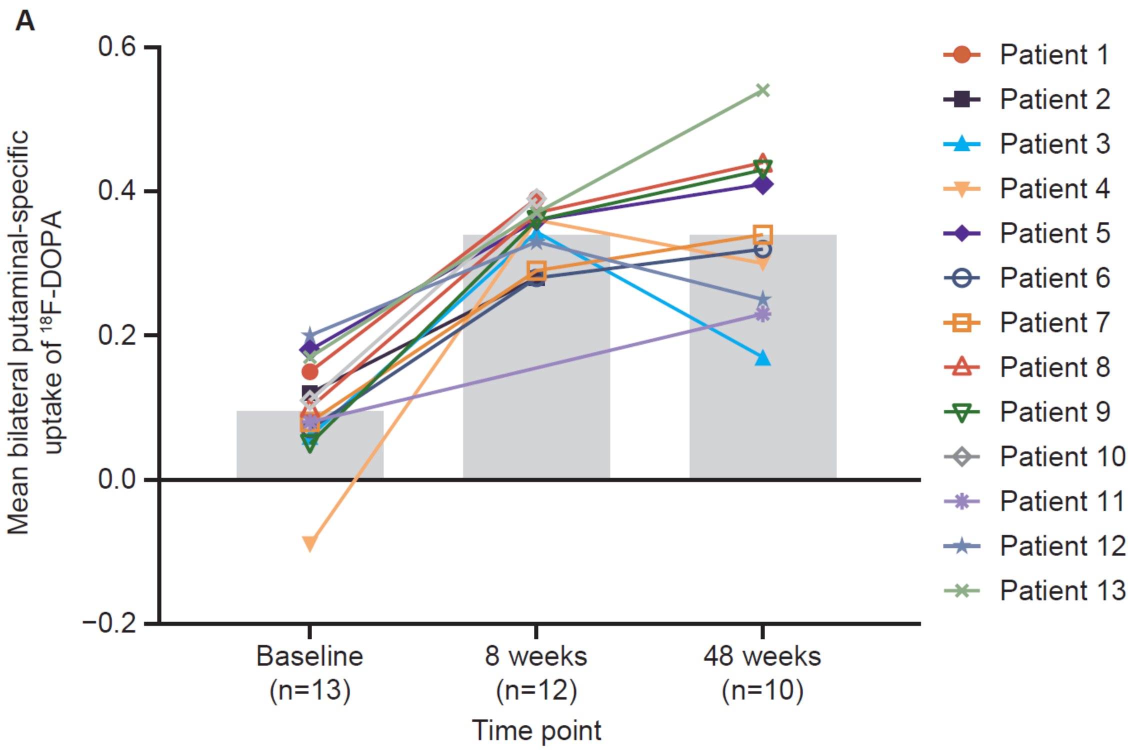

B

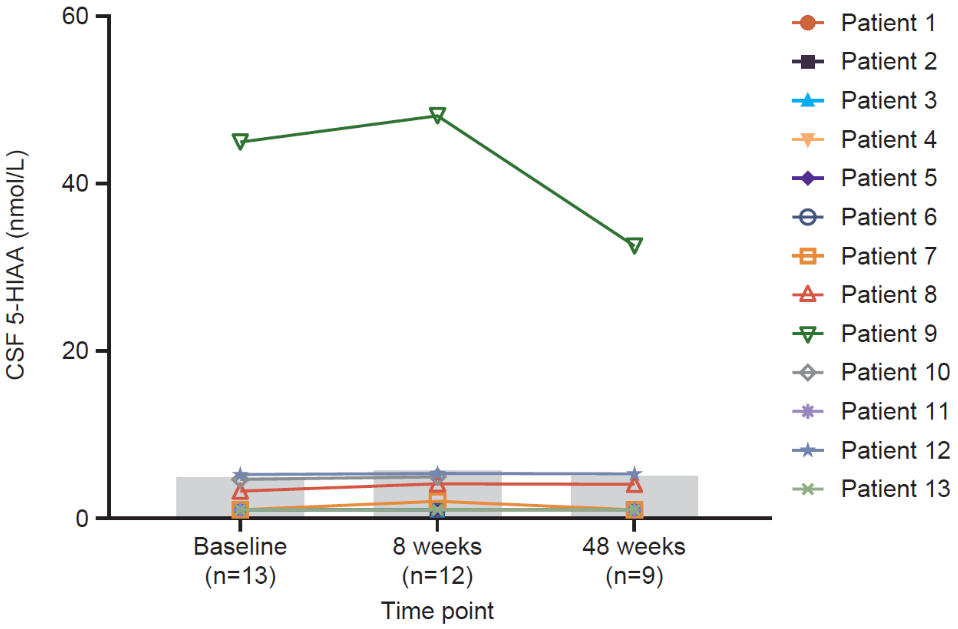

C

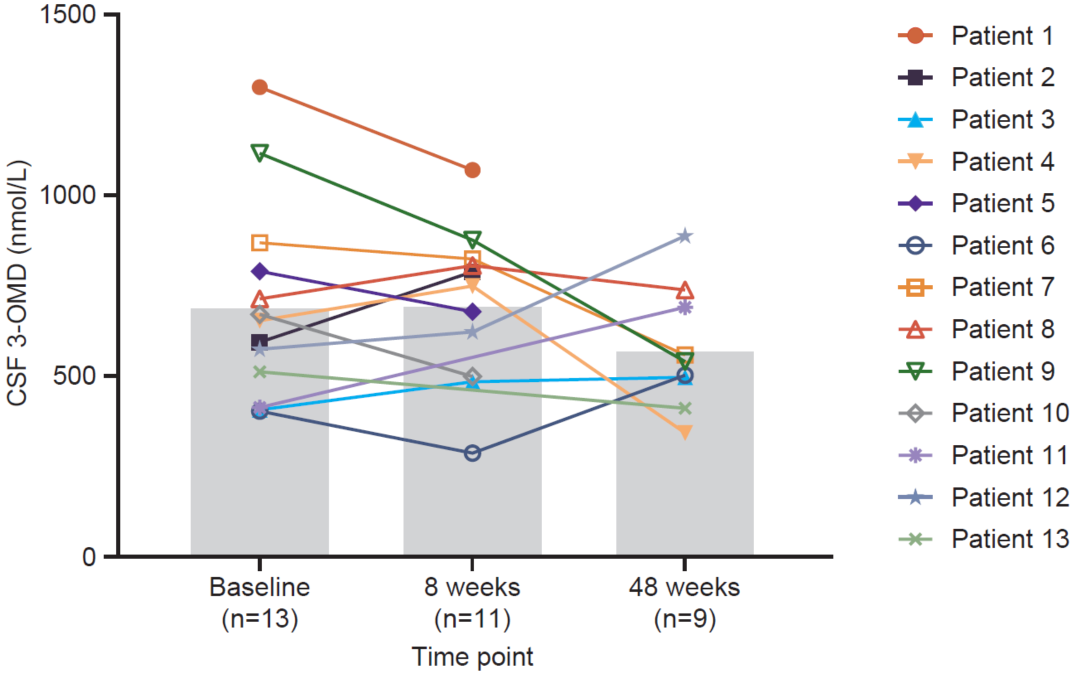

The grey bars show the mean level at each time point. (A) The following mean bilateral putaminal-specific uptake of ^{18}F-DOPA values were recorded: baseline, 0.098 (SD: 0.074); 8 weeks, 0.343 (SD: 0.040) ($p<0.0001$ *vs* baseline); 48 weeks, 0.343 (SD:

0.113) (p<0.0001 *vs* baseline). (B) Values noted as <2.0 nmol/L are shown here as 1.0 nmol/L. The following mean CSF 5-HIAA levels were recorded: baseline, 5.2 (SD: 12.1) nmol/L; 8 weeks, 6.0 (SD: 13.4) nmol/L (p=0.1103 *vs* baseline); 48 weeks, 5.3 (SD: 10.3) nmol/L (p=0.3858 *vs* baseline). (C) The following mean CSF 3-OMD levels were recorded: baseline, 693.1 (SD: 272.5) nmol/L; 8 weeks, 698.3 (SD: 216.6) nmol/L (p=0.4260 *vs* baseline); 48 weeks, 573.9 (SD: 170.2) nmol/L (p=0.5942 *vs* baseline). ^{18}F-DOPA=L-6-[^{18}F] fluoro-3,4-dihydroxyphenylalanine. 3-OMD, 3-*O*-methyldopa. 5-HIAA, 5-hydroxyindoleacetic acid. CSF, cerebrospinal fluid. SD, standard deviation.
